# Construction of recombinant pseudorabies virus expressing PCV2 Cap, PCV3 Cap, and IL-4: investigation of their biological characteristics and immunogenicity

**DOI:** 10.3389/fimmu.2024.1339387

**Published:** 2024-03-20

**Authors:** Yanting Yang, Zhiwen Xu, Qian Tao, Lei Xu, Sirui Gu, Yao Huang, Zheyan Liu, Yang Zhang, Jianhua Wen, Siyuan Lai, Ling Zhu

**Affiliations:** College of Veterinary Medicine, Sichuan Agricultural University, Chengdu, China

**Keywords:** porcine circovirus, Cap protein, porcine pseudorabies virus, immunogenicity, viral challenge protection

## Abstract

**Background:**

Porcine circovirus type 2 (PCV2) is a globally prevalent and recurrent pathogen that primarily causes slow growth and immunosuppression in pigs. Porcine circovirus type 3 (PCV3), a recently discovered virus, commonly leads to reproductive disorders in pigs and has been extensively disseminated worldwide. Infection with a single PCV subtype alone does not induce severe porcine circovirus-associated diseases (PCVD), whereas concurrent co-infection with PCV2 and PCV3 exacerbates the clinical manifestations. Pseudorabies (PR), a highly contagious disease in pigs, pose a significant threat to the swine industry in China.

**Methods:**

In this study, recombinant strains named rPRV-2Cap/3Cap and rPRV-2Cap/3Cap/IL4 was constructed by using a variant strain XJ of pseudorabies virus (PRV) as the parental strain, with the TK/gE/gI genes deleted and simultaneous expression of PCV2 Cap, PCV3 Cap, and IL-4. The two recombinant strains obtained by CRISPR/Cas gE gene editing technology and homologous recombination technology has genetic stability in baby hamster Syrian kidney-21 (BHK-21) cells and is safe to mice.

**Results:**

rPRV-2Cap/3Cap and rPRV-2Cap/3Cap/IL4 exhibited good safety and immunogenicity in mice, inducing high levels of antibodies, demonstrated 100% protection against the PRV challenge in mice, reduced viral loads and mitigated pathological changes in the heart, lungs, spleen, and lymph nodes during PCV2 challenge. Moreover, the recombinant viruses with the addition of IL-4 as a molecular adjuvant outperformed the non-addition group in most indicators.

**Conclusion:**

rPRV-2Cap/3Cap and rPRV-2Cap/3Cap/IL4 hold promise as recombinant vaccines for the simultaneous prevention of PCV2, PCV3, and PRV, while IL-4, as a vaccine molecular adjuvant, effectively enhances the immune response of the vaccine.

## Introduction

Porcine circovirus (PCV) is a non-enveloped, circular, single-stranded DNA virus belonging to *Circoviridae* and the genus *Circovirus* (1). Currently, four genotypes of PCV have been identified, namely PCV1, PCV2, PCV3, and PCV4 (2). PCV2 was initially identified in, 1998. Its complete genomic length measures 1.76 kilobases (kb) (3). To this day, PCV2 remains a globally prevalent pathogen, causing

many symptoms in infected pigs, including multi-systemic failure, enteritis, pneumonia, and reproductive disorders. These diseases are collectively referred to as Porcine Circovirus Disease (PCVD) or Porcine Circovirus-Associated Diseases (PCVAD) (4). In, 2016, the United States detected a PCV3 variant with a complete length of 2 kb for the first time. This variant’s Rep and Cap proteins exhibited only 31-48% homology with PCV1 and PCV2 (5). PCV3 has been reported to be prevalent in multiple countries (5, 6), primarily causing reproductive disorders, skin diseases, and multi-systemic inflammation in pigs (7, 8). Recent research has revealed that co-infection of PCV2 and PCV3 often accompanies viral viremia and the manifestation of PCVAD symptoms (9).

Cap protein is the only structural protein of PCV2 and PCV3 and serves as their primary immunogenic protein. It contains the major antigenic determinants of the virus, stimulating the production of specific neutralizing antibodies in the organism (10, 11). The Cap protein exhibits polymorphism and plays a significant role in the process of PCV attachment while also exerting a certain influence on assembly dynamics and structural stability. Furthermore, the Cap protein harbors the primary protective antigenic epitopes of PCV, capable of eliciting an immune response in the organism and thus serving as an ideal antigen for developing genetic engineering vaccines (10). By conducting a homology analysis on the genetic sequences of PCV2 and PCV3, it was found that the homology between the two serotypes of porcine circovirus was relatively low. The homology of their Cap proteins was only 26%, and no cross-protection was observed (12). Therefore, the Cap protein represents a crucial choice for developing PCV2 and PCV3 vaccines.

Pseudorabies virus (PRV), belonging to the family *Herpesviridae*, subfamily *Alphaherpesvirinae*, and genus *Varicellovirus*, is known to cause clinical symptoms in infected pigs. These symptoms primarily include fatal encephalitis in neonatal piglets, characterized by neurological disorders such as tremors, motor disturbances, and lethargy, as well as slow growth in growing pigs and reproductive disorders in sows. The disease has a high morbidity and mortality rate (13). The pseudorabies virus (PRV), deleting virulence genes such as gE, gI, gG, and TK, has been rendered avirulent and is a valuable tool for efficiently expressing exogenous antigens. As a result, it has been widely employed in preparing various animal vaccines (14). Various recombinant vaccines have utilized pseudorabies virus (PRV) as a live vector to express the major immunogenic proteins of other pathogens. For instance, the spike (S) protein of porcine deltacoronavirus (PDCoV), VP2 protein of porcine circovirus (PCV), and GP5 and M proteins of porcine reproductive and respiratory syndrome virus (PRRSV) has been expressed using PRV as a vector (15–17).

IL-4, a hallmark cytokine of type 2 immune responses, is produced by various cell types, including T cells, innate lymphoid cells, and bone marrow-derived cells (such as basophils and mast cells) (18). It can modulate humoral and cellular immune responses, such as the production, class switching, and secretion of immunoglobulins (19). IL-4, while enhancing the organism’s immune response, possesses a high degree of safety, making it an effective vaccine adjuvant.

Previous studies have indicated that vaccines containing cytokines and protective antigenic proteins can induce superior immune responses compared to antigens alone (20–23). In this study, we engineered a novel recombinant PRV strain lacking the gE, gI, and TK proteins, which co-expressed the PCV2 Cap protein, PCV3 Cap protein, and IL-4, attempting to generate a recombinant vaccine that protects against PCV2, PCV3 and PRV simultaneously. We evaluated the safety of this strain and assessed its ability to induce humoral and cellular immune responses in mice. Our research findings provide valuable evidence for future vaccine development targeting PCV2, PCV3, and PRV strains. Additionally, we evaluated the efficacy of IL-4 as an immune adjuvant, demonstrating its capability to enhance protective immune responses against the recombinant PRV in mice.

## Materials and methods

### Cells, viruses, pathogens, plasmids, antibodies and mice

The PRV-XJ-ΔTK strain was constructed and preserved by the Animal Biotechnology Center, College of Veterinary Medicine, Sichuan Agricultural University (Chengdu, China), and propagated in BHK-21 cells. BHK-21 cells were cultured in DMEM (Gibco) supplemented with 10% fetal bovine serum (BI) at 37°C with 5% CO_2_. The lentiCRISPR v2-gE plasmid expressing PRV gE sgRNA (GGGCAGGAACGTCCAGATCC) was constructed and preserved in our laboratory. The PCV2 Cap protein, PCV3 Cap protein, and their respective rabbit polyclonal antibodies were prepared and preserved by the Animal Biotechnology Center, Sichuan Agricultural University. 6-week-old female BALB/c mice were purchased from Beijing Huafukang Biotechnology Co with SPF breeding grade.

### Construction of recombinant transfer plasmid

The pEGFP-gI28k eukaryotic expression plasmid was sent to Genscript Biotech Corporation in Nanjing. Based on the sequenced PCV2MY-202011 (belongs to PCV2d, GenBank: OP055737.1), PCV3DY-202010 (belongs to PCV3a, GenBank: OP066315.1), and IL-4 (GenBank: HQ236500.1) obtained from our laboratory, synthetic PCV2 ORF2, PCV3 ORF2, and IL-4 genes were generated. T2A self-cleaving peptide was inserted between EGFP and 2Cap, F2A self-cleaving peptide was inserted between 2Cap and 3Cap, and P2A self-cleaving peptide was inserted between 3Cap and IL-4. The entire sequence was then inserted into the pEGFP-gI28k vector, generating pEGFP-gI28k-2Cap-3Cap and pEGFP-gI28k-2Cap-3Cap-IL4 transfer vectors.

pEGFP-gI28k-2Cap-3Cap, pEGFP-gI28k-2Cap-3Cap-IL4 plasmids, and lentiCRISPR v2-gE plasmid were extracted using the appropriate Mini Plasmid Extraction Kit (OMEGA, USA).

### Construction of the recombinant virus

Following the instructions provided in the LipofectamineTM, 3000 Reagent kit (Invitrogen, USA), pEGFP-gI28k-2Cap-3Cap and pEGFP-gI28k-2Cap-3Cap-IL4 were separately co-transfected with lentiCRISPR v2-gE into BHK-21 cells. After 24 h, 10 μl of PRV-XJ-ΔTK virus was added. When approximately 80% cytopathic effect was observed, the cells were subjected to three freeze-thaw cycles at -80°C to collect the virus. The recombinant virus was purified using the limited dilution method in a 96-well plate and the plaque purification method in a 6-well plate.

### PCR identification ofrPRV-2Cap/3Cap and rPRV-2Cap/3Cap/IL4

Following the instructions provided by the DNA extraction kit manual, viral DNA was extracted as a template. Specific primers were employed to detect the absence of TK and gE genes, as well as the insertion of PCV2 Cap, PCV3 Cap, and IL-4 genes within the rPRV-2Cap/3Cap and rPRV-2Cap/3Cap/IL4 DNA samples. PRV XJ DNA was utilized as the positive control, while a template lacking DNA served as the negative control. Primers are shown in **Table 1**.

**Table 1:** PCR primer.

Target gene	primer sequence(5’-3’)
TK	GATGACATACACATGGCTTTATACGCGCC
	TCACCGCCGCGGCCCGGCGACGTACTC
gE	ATCTGGACGTTCCTGCCC
	GTAGATGCAGGGCTCGTACA
PCV2 Cap	AATGGCATCTTCAACACCCG
	AGACCCCCCACTTAACCCT
PCV3 Cap	ACATACTACACAAAGAAATACTCAACC
	ATTCGTTACAAGTCCGTTCTC
IL-4	ACACAAGTGCGACATCACCT
	GGCTTCATGCACAGAACAGG

### Western blotting

Recombinant viruses rPRV-2Cap/3Cap and rPRV-2Cap/3Cap/IL4 were used to infect BHK-21 cells. Subsequently, the cells were lysed using RIPA (Radioimmunoprecipitation Assay) buffer containing 1mM phenylmethanesulfonyl fluoride (PMSF). Proteins were extracted and separated by 10% SDS-PAGE gel electrophoresis, then transferred onto a PVDF membrane. The membrane was then blocked with 5% skimmed milk (prepared in PBS) at room temperature for 2 h. After washing with PBST (PBS containing 0.5% Tween 20), the membrane was incubated overnight at 4°C with primary antibodies. The primary antibodies used were: PCV2 Cap rabbit polyclonal antibody (from our lab) diluted 1:200, PCV3 Cap rabbit polyclonal antibody (from our lab) diluted 1:200, IL-4 antibody (ab84269, Abcam) diluted 1:5000, and reference gene GAPDH antibody (ab8245, Abcam) diluted 1:5000. After washing, the membrane was incubated at room temperature for 2 h with secondary antibodies (horseradish peroxidase (HRP)-conjugated IgG antibodies) (ab205718/ab205719, Abcam) diluted 1:5000. Finally, the membrane was treated with Clarity Western ECL substrate (170-5061, Bio-Rad) and imaged using a chemiluminescence imaging system.

### Immunofluorescence assay

When BHK-21 cells reached a dense monolayer growth in a 24-well plate, they were inoculated with 5 μl of rPRV-2Cap/3Cap, rPRV-2Cap/3Cap/IL4, and PRV XJ. Once the cells showed pathological changes but were not completely detached, the supernatant was discarded, and the cells were fixed with 4% paraformaldehyde for 30 min, permeabilized with 30% acetone for 20 min, and then sealed with 5% BSA for 60 min. The cells were then incubated overnight with PCV2 Cap polyclonal antibody (1:200) as the primary antibody, followed by incubation for 2 h in darkness with FITC-conjugated goat anti-rabbit IgG (1:300) (SA00013-3; Proteintech) as the secondary antibody. Subsequently, the cells were incubated in darkness for 15 min with DAPI staining reagent. Each step required washing the cells thrice with PBST, lasting 5 min. The fluorescence lesions were observed under a fluorescence microscope.

### Plaque assays

BHK-21 cells were seeded into a 6-well plate at a cell density of approximately 2×10^6^-4×10^6^ cells/ml. After gradient dilution (10^-2^-10^-7^), recombinant viruses rPRV-2Cap/3Cap, rPRV-2Cap/3Cap/IL4, and parental strain PRV XJ were inoculated into each well with 200 μl of viral suspension, and the plate was placed in a cell culture incubator (37°C, 5% CO_2_) for 1 h to allow adsorption. The liquid in the wells was discarded, and the cells were washed twice with PBS. A mixture of 1% methylcellulose and 2×DMEM (1:1) was added to each well, with a volume of 2 ml per well. After incubation at 37°C for 48-72 h, the cells were stained with 5% (w/v) crystal violet for 30 min, then rinsed with distilled water, and the number and area of plaques were automatically determined using IPP6.0 software.

### Replication kinetics of recombinant virus

To analyze the growth characteristics of the recombinant viruses, BHK-21 cells cultured in 12-well plates were infected with 10^4^ TCID_50_ (tissue culture infective dose) of rPRV-2Cap/3Cap, rPRV-2Cap/3Cap/IL4, and PRV XJ, respectively, with 100 μl per well. The plates were placed in a cell culture incubator (37°C, 5% CO_2_) for 1 h to allow adsorption. After washing the cells thrice with PBS, 1 ml of DMEM cell maintenance medium was added to each well. At 0, 2, 4, 6, 8, 10, 12, 24, 36, 48, 60, and 72 h, all cells and supernatants in each well were collected, with three samples taken at each time point as replicates. The 50% tissue culture infective dose (TCID_50_) was calculated using the Reed-Muench method, and a one-step growth curve of the virus was plotted.

### Transmission electron microscopic observation of virions

BHK-21 cells were subcultured into a 6-well plate. When the cell density reached 80%, each well was inoculated with 10^4^ TCID_50_ (tissue culture infective dose) of rPRV-2Cap/3Cap, rPRV-2Cap/3Cap/IL4, and PRV XJ, respectively. The plate was then placed in a cell culture incubator (37°C, 5% CO_2_) for 1 h to allow adsorption. The liquid in the wells was discarded, and 2 ml of DMEM containing 2% FBS was added. After 24 h, the cells in the wells were collected and treated with 0.5% and 3% glutaraldehyde fixatives, respectively, and then sent to Chengdu Rilai Biotechnology Co., Ltd. for further processing. The viral images were acquired using a JEM-1400plus transmission electron microscope.

### Genetic stability

To assess the genetic stability of recombinant viruses rPRV-2Cap/3Cap and rPRV-2Cap/3Cap/IL4, they were passaged 21 times in BHK-21 cells. Samples from passages F1, F5, F10, F15 and F21 were taken, and PCR analysis was performed to confirm the deletion of the TK and gE genes and the insertion of PCV2 Cap, PCV3 Cap, and IL-4 genes. PRV XJ DNA was used as a positive control, while a template without DNA was a negative control.

### Safety and immunogenicity assessment in mice

This study randomly selected 52 six-week-old female BALB/c mice and divided them into 7 groups. The first 6 groups served as experimental groups, with 8 mice in each group, and were injected with viral suspensions of rPRV-2Cap/3Cap or rPRV-2Cap/3Cap/IL4 at doses of 200 ml 10^7^ TCID_50_, 200 ml 10^6^ TCID_50_, and 200 ml 10^5^ TCID_50_, respectively. The 7th group consisted of 4 mice serving as the control group, which received an intramuscular injection of 0.2 ml DMEM. The mice were observed continuously for 15 d, and their survival status was recorded. The survival curves of mice in different groups were plotted using GraphPad Prism 9 software. At 3 d postvaccination, the brains were collected for H&E staining.

### Mouse immunization and challenge experiment

**Figure 1:**
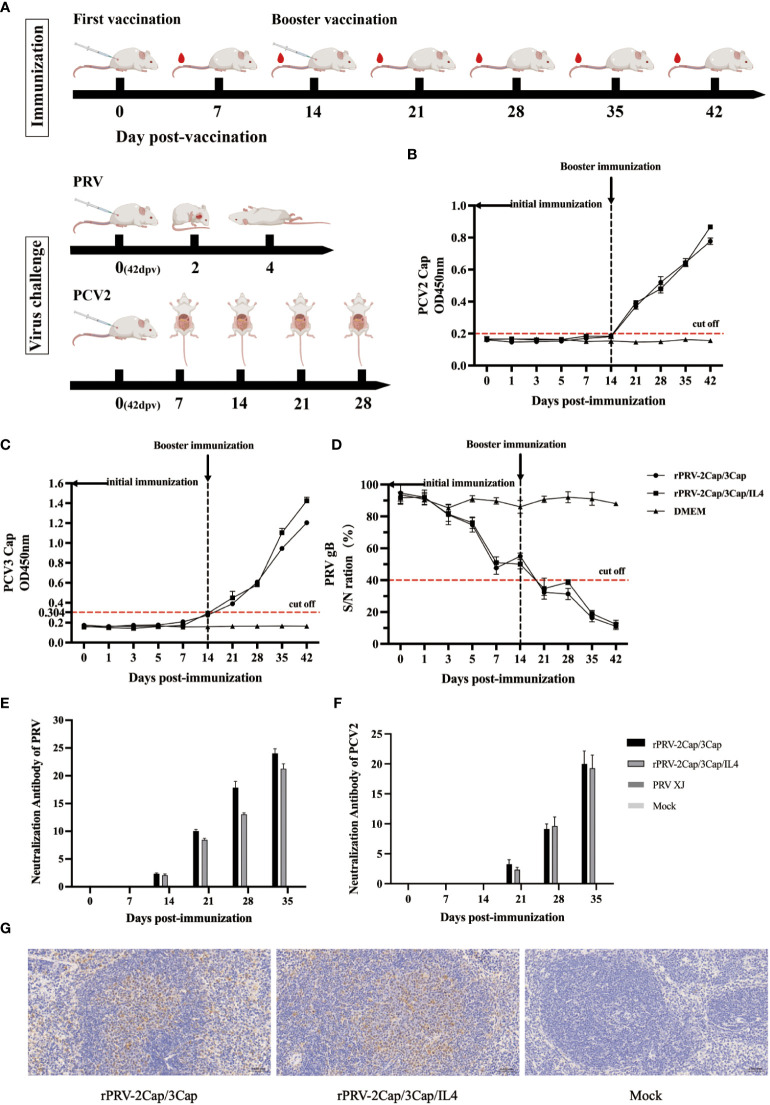
Humoral immune responses after muscular immunization with recombinant virus. **(A)** Graphical representation of immunization/virus challenge experiment. **(B)** PCV2 Cap-specific antibodies in the serum from 0 to 42 dpv after vaccination were measured by ELISA. (n=3) **(C)** PCV3 Cap-specific antibodies were measured by ELISA. (n=3) **(D)** The levels of PRV gB-specific antibodies were measured by blocking ELISA. (n=3) **(E)** Neutralizing antibody titers against the PRV-XJ strain. (n=3) **(F)** Neutralizing antibody titers against the PCV2-SC strain. (n=3) **(G)** Immunohistochemical results of spleen tissues in mice (200X).

The immune/viral challenge experiment is graphically represented in **Figure 1A**. Female BALB/c mice at 6 weeks of age were randomly divided into three groups. The negative control group received intramuscular inoculation of DMEM (n = 30/group), while experimental Group A (rPRV-2Cap/3Cap group) (n = 30/group) was inoculated with 200 ml 10^6^ TCID_50_ of rPRV-2Cap/3Cap, and experimental Group B (rPRV-2Cap/3Cap/IL4 group) (n = 30/group) was inoculated with 200 ml 10^6^ TCID_50_ of rPRV-2Cap/3Cap/IL4. Blood samples were collected from the tail vein of mice at 0, 1, 3, 5, 7, 14, 21, 28, 35, and 42 d, and the sera were separated and stored at -80°C. Splenocytes were isolated from mice on D 14 and D 28.

In the 8th week after the initial immunization, each group of mice received an intramuscular injection of 200 μl of 10^5^ TCID_50_ of PCV2-SC (belongs to PCV2b) (n = 12/group). On d 7, 14, 21, and 28 after PCV2 inoculation, heart, liver, spleen, lung, kidney, brain, intestine, mesenteric lymph node specimens, and serum were collected from each group (n=3) for PCV2 viremia determination, PCV2 viral load analysis, and histopathological observations.

Additionally, each group of mice was challenged with 200 μl of 10^5^ TCID_50_ of PRV-XJ (n = 10/group) via intramuscular injection. All mice were monitored within 15 d after the challenge, and brain tissues were collected for histopathological examination. Surviving mice were euthanized at the end of the 15-d period.

All experimental procedures were reviewed and approved by the Sichuan Agriculture University Animal Care and Use Committee (license number SCXK [Sichuan], 2013-0001).

### ELISA for specific antibodies and cytokines

The ID.vet gE antibody detection kit was employed in this study to assess the levels of PRV gE antibodies in mouse serum. For the detection of PCV2 Cap antibodies, the operation was carried out following the instructions provided by Shenzhen Zhenrui Biotechnology Co., Ltd. for the porcine circovirus antibody detection kit. In this case, HRP-labeled rabbit anti-mouse secondary antibodies were used instead of the enzyme-labeled antibodies provided in the original kit. The PCV3 Cap antibody detection operation was conducted according to the instructions provided by Shanghai Keaibo Biotechnology Co., Ltd. for the mouse circovirus type 3 detection kit. The secretion levels of IL-6 and IFN-γ were measured on d 14 and 28 post-immunization using the mouse-specific IFN-γ and IL-6 cytokine detection kits provided by Ximbio Biotechnology Co., Ltd.

### Murine splenic lymphocyte proliferation test

After 14 and 28 d post-immunization with recombinant viruses, spleen cells were made isotonic in, 1640 medium and seeded in a 96-well plate at a density of 5×10^6^ cells/ml (100 μl per well). The proliferation of spleen cells from each group of mice (n=3) was measured under four conditions: (1) purified PCV2 Cap protein (10 µg/ml), (2) purified PCV3 Cap protein (10 µg/ml), (3) ConA (A phytohemagglutinin with potent mitogenic capacity, which can be used as a positive standard for stimulating splenic lymphocyte proliferation (24)) (10 µg/ml), and (4) 1640 medium. Each condition was performed in triplicate, and the average was calculated. After 72 h of incubation in the wells, the absorbance values were measured using a CCK-8 assay kit (Beyotime, China) to detect each well’s optical density (OD) at 450 nm wavelength. Based on these data, stimulation indices (SI) were calculated to assess the proliferation of spleen lymphocytes. The formula for calculating the stimulation index is as follows: SI = (OD value of the immunized group - OD value of the blank control group)/(OD value of the negative control group - OD value of the blank control group).

### Flow cytometry analysis of CD3/CD4/CD8 murine splenic lymphocytes

Splenocytes were isolated from mouse spleens in the second week after enhancing immunity (n=3). The splenocytes were obtained from the spleen samples by passing them through a 100-micron cell strainer. Subsequently, the spleen cells were lysed using ammonium chloride-potassium (ACK) lysis buffer at 4°C for 5 min. Approximately 10^6^ splenic lymphocytes were prepared and stained with fluorescein isothiocyanate (FITC)-conjugated anti-mouse CD3 antibody, allophycocyanin (APC)-conjugated anti-mouse CD4 antibody, and phycoerythrin (PE)-conjugated anti-mouse CD8a antibody (100204, 100412, and, 100708, BioLegend) at a dilution of 1:250. The staining was performed in the dark at 4°C for 30 min. The stained samples were analyzed using a BD FACSVerse flow cytometer, and data collection and analysis were performed using Flowjo 10 software.

### Neutralizing antibody assay in mice

Serum samples from mice in the immunized and control groups (n=3), collected at 7, 14, 28, and 35 d post-vaccination, were subjected to heat inactivation at 56°C in a water bath for 30 min. The samples were then diluted with DMEM in a two-fold serial dilution (1:2n). Each was mixed with a 200 TCID_50_ PCV2 virus solution and incubated at 37°C in a 5% CO_2_ incubator for 1 h. Subsequently, the mixture was added to 96-well plates lined with PK-15 cells and cultured. After 72 h, the cells were washed twice with phosphate-buffered saline (PBS). Subsequently, the cells were fixed with a mixture of ice-cold acetone/methanol (1:1) at -20°C for 20 min, followed by blocking with 3% bovine serum albumin (BSA) (diluted with PBS) at room temperature for 1 h. The cells were then incubated with a 1:100 dilution of PCV2-positive serum (from our lab) at 4°C overnight. After washing the plates, staining was performed using Cy3-labeled goat anti-rabbit IgG (1:200) at 37°C for 1 h, and the samples were observed and counted under a fluorescence microscope. Finally, the serum antibody titers were calculated using the Reed-Muench method.

Similarly, diluted sera were mixed with an equal volume of 100 PFU PRV-XJ at 37°C for 1 h. Following the neutralization reaction, the virus-serum mixture was inoculated onto 80% confluent BHK-21 cells in a 6-well plate and incubated at 37°C until cytopathic effects (CPE) appeared. Mouse serum-negative samples were used as controls. The 6-well plates were stained using crystal violet staining solution and imaged using IPP6.0 software to read the number and area of plaques automatically. Serum neutralization titers were assessed using the plaque reduction assay, where the serum dilution resulting in a 50% reduction of plaque count was employed as the neutralization titer for the sample. The plaque reduction assay utilized a proportional calculation approach.

### Immunohistochemical

Using rabbit-derived polyclonal antibodies against PCV2 Cap (1:100, from our lab) as the primary antibody and goat anti-rabbit IgG (1:200) as the secondary antibody, immunohistochemical analysis was performed to investigate the colonization of recombinant viruses rPRV-2Cap/3Cap and rPRV-2Cap/3Cap/IL4 in mouse spleens.

### PCV2 viral load in serum and tissues

After DNA extraction, quantitative detection of PCV2 DNA in serum, heart, lung, liver, spleen, kidney, small intestine, mesenteric lymph nodes, and brain tissues was performed using a real-time fluorescence quantitative PCR (qPCR) assay (25).

### Histopathology assay

The PRV-challenged mice’s lungs, spleen, heart, and mesenteric lymph nodes were fixed using a 4% paraformaldehyde solution. The fixed samples were dehydrated, embedded in paraffin, and cut into 4 μm-thick sections. The sections were stained with hematoxylin and eosin after mounting.

### Statistical analysis

Statistical analysis was performed and histogram were drawn using GraphPad PrismTM 8.0 (GraphPad Soft- ware, USA), Paired student t-test, and one-way ANOVA was used to test differences between different groups. P values< 0.05 were considered significant.

## Results

### Construction of recombinant viruses

**Figure 2:**
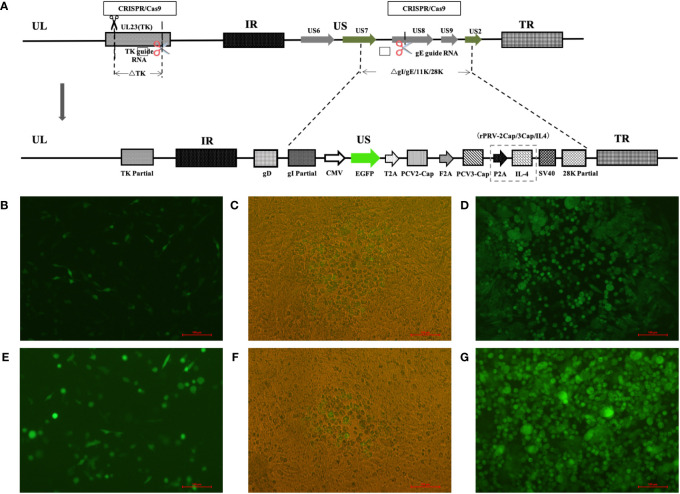
Construction of the recombinant viruses rPRV-2Cap/3Cap and rPRV-2Cap/3Cap/IL4. **(A)** Schematic diagram of the recombinant pseudorabies virus (PRV) development strategy. **(B)** Fluorogram of eukaryotic transfer vector pEGFP-gI28K-2Cap-3Cap transfection. **(C)** The first observation of lesions in rPRV-2Cap/3Cap. **(D)** Plaque purification for rPRV-2Cap/3Cap. Green plaques are the BHK-21 cells infected with recombinant viruses. **(E)** Fluorogram of eukaryotic transfer vector pEGFP-gI28K-2Cap-3Cap-IL4 transfection. **(F)** The first observation of lesions in rPRV-2Cap/3Cap/IL4. **(G)** Plaque purification for rPRV-2Cap/3Cap/IL4.

Using CRISPR/Cas9 technology and homologous recombination, recombinant pseudorabies viruses (rPRV-2Cap/3Cap and rPRV-2Cap/3Cap/IL4) expressing PCV2 Cap, PCV3 Cap (and IL-4) proteins were constructed (**Figure 2A**). Under an inverted microscope (Nikon, Japan), green fluorescent-labeled recombinant proteins could be observed in BHK-21 cells 24 h after transfection (**Figure 2B**). The first cytopathic effect (CPE) caused by rPRV-2Cap/3Cap was observed three d after the addition of the PRV TK gene-deleted strain (**Figure 2C**), along with the purified recombinant virus (**Figure 2D**).Similarly, rPRV-2Cap/3Cap/IL4 was obtained (**Figures 2E–G**).

### Characterization of recombinant viruses

**Figure 3:**
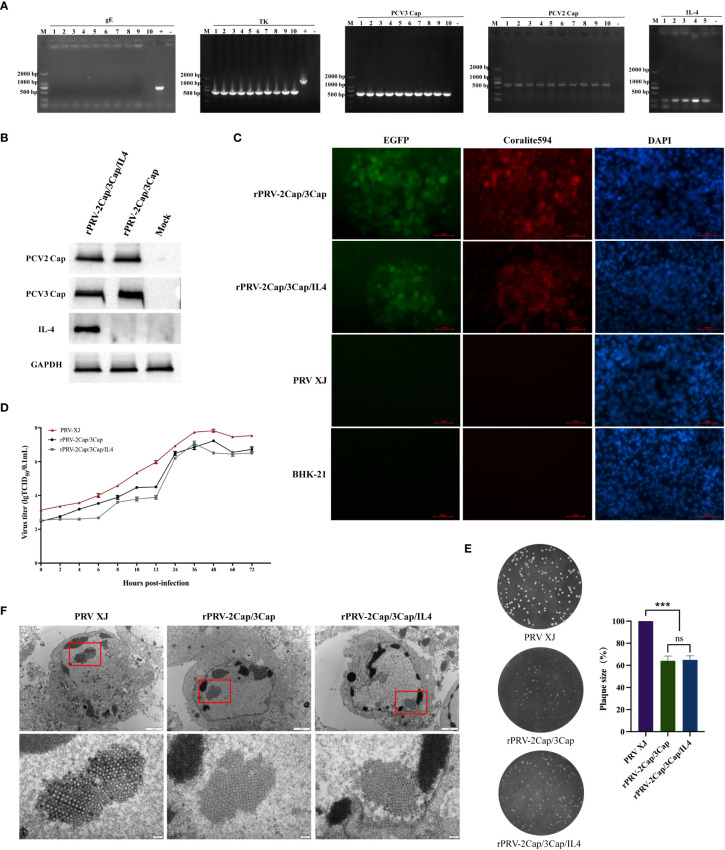
Characterization of recombinant viruses rPRV-2Cap/3Cap and rPRV-2Cap/3Cap/IL4. **(A)** PCR identification of the deletion of TK, gE, and the insertion of PCV2-Cap, PCV3-Cap, and IL-4 in recombinant virus (1-5 are rPRV-2Cap/3Cap/IL4 F1, F5, F10, F15, and F21. 6-10 are rPRV-2Cap/3Cap F1, F5, F10, F15, and F21), with the parental strain PRV-XJ (XJ) as a control. **(B)** Western blotting of protein in BHK-21 cells infected with the recombinant viruses. Mock, BHK-21 cell lysis products. **(C)** Immunofluorescence assay (IFA) detection of PCV2-Cap protein expression. Red, PCV2Cap protein bound with anti-PCV2Cap antibody and labeled by Corallite 594; green, EGFP tag in recombinant virus; blue, 4’,6-diamidino-2-phenylindole (DAPI)-stained BHK- 21 cell nucleus. **(D)** One-step growth curve of the recombinant strains and parent strain in BHK-21. (n=3) **(E)** Comparison of the plaque size of BHK-21 cells infected with the recombinant and parent strains. (n=3) **(F)** Transmission electron microscopy analysis of BHK-21 cells infected with the recombinant and parent strains. Data are presented as mean ± SD (n = 3); ns, not significant; ***, *P*<0.01.

rPRV-2Cap/3Cap and rPRV-2Cap/3Cap/IL4 strains were consecutively cultured for 21 generations. Viral fluids from the 1st, 5th, 10th, 15th, and 21st generations were collected. The PRV TK, gE, PCV2-Cap, PCV3-Cap, and IL-4 genes were detected by PCR analysis using recombinant viral and parental strain DNA. The results showed stable expression of the inserted genes, negative expression of the gE gene, and shortening of the TK gene due to CRISPR-Cas9 cleavage (**Figure 3A**). Western blot analysis was conducted to examine the expression of PCV2-Cap, PCV3-Cap, and IL-4 proteins in BHK-21 cells infected with rPRV-2Cap/3Cap and rPRV-2Cap/3Cap/IL4. Specific bands were detected in cells infected with recombinant viruses compared to BHK-21 negative cells (**Figure 3B**). Indirect immunofluorescence assay (IFA) revealed specific red fluorescence signals for PCV2-Cap protein in BHK-21 cells infected with rPRV-2Cap/3Cap and rPRV-2Cap/3Cap/IL4, using rabbit anti-PCV2-Cap polyclonal antibodies. No red fluorescence signal was observed in PRV XJ-infected BHK-21 cells in the control group or negative control cells (**Figure 3C**).

To assess the impact of exogenous fragment insertion, the one-step growth curves of PRV-XJ, rPRV-2Cap/3Cap, and rPRV-2Cap/3Cap/IL4 were analyzed using the Reed-Muench method, and the viral plaque-forming ability was examined through plaque assays. The curves demonstrated that PRV XJ, rPRV-2Cap/3Cap, and rPRV-2Cap/3Cap/IL4 exhibited similar growth trends with comparable growth kinetics and viral titers. However, the viral titer of PRV XJ was higher than that of rPRV-2Cap/3Cap and rPRV-2Cap/3Cap/IL4 (**Figure 3D**). The plaque sizes of rPRV-2Cap/3Cap and rPRV-2Cap/3Cap/IL4 were similar but significantly smaller than those of the parental strain PRV XJ (**Figure 3E**). Transmission electron microscopy observations revealed that the viral particles of rPRV-2Cap/3Cap and rPRV-2Cap/3Cap/IL4 were similar to those of PRV XJ, with no significant differences observed (**Figure 3F**).

### rPRV-2Cap/3Cap and rPRV-2Cap/3Cap/IL4 is safe for mice

**Figure 4:**
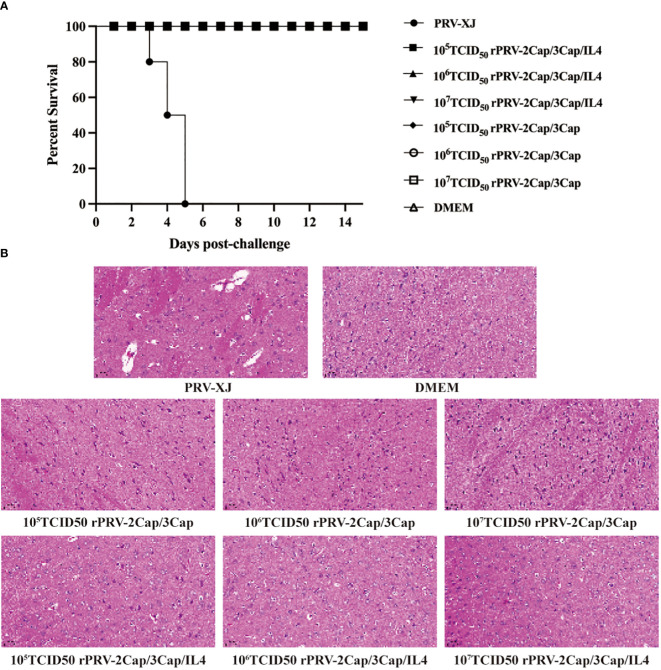
The safety assessment of the recombinant virus. **(A)** Survival curves of mice infected with the parent strain and different titers of the recombinant strain. (n=10) **(B)** Pathological observation of H&E staining in brain tissue of mice infected with the PRV-XJ, DMEM, or different titers of the recombinant strain. The PRV-XJ group shows nuclear cleavage, vacuolate neuronal degeneration, and neuron phagocytosis.

The safety of the recombinant viruses was evaluated using mice. The mice in the PRV-XJ group exhibited pruritus and scratching symptoms 3 d after infection and died within 5 d. In contrast, mice in the rPRV-2Cap/3Cap and rPRV-2Cap/3Cap/IL4 groups, after being inoculated with 10^5^, 10^6^, and 10^7^ 50% TCID_50_, did not show any clinical symptoms and survived until the end of the observation period (**Figure 4A**). Histopathological changes in mouse brain tissues were analyzed using HE staining. PRV-XJ infection resulted in neuronal damage, including vacuolar degeneration, neuronal phagocytosis, and nuclear division, while no histopathological changes were observed in the tissues of mice infected with the recombinant strains (**Figure 4B**).

### Serological immunogenicity

**Figure 1A** depicts the process of mouse immunization. ELISA assay detected specific antibodies against PRV-gB, PCV2-Cap, and PCV3-Cap generated by immunized mice. Compared to the DMEM group, recombinant vaccines rPRV-2Cap/3Cap and rPRV-2Cap/3Cap/IL4 induced the production of specific antibodies against PCV2 Cap, PCV3 Cap, and PRV gB proteins starting at d 21 post-immunization (**Figures 1B–D**, respectively). Furthermore, at d 42 post-immunization, mice in the rPRV-2Cap/3Cap/IL4 group exhibited significantly higher levels of PCV2 Cap and PCV3 Cap antibodies compared to the rPRV-2Cap/3Cap group (*P*<0.01), indicating that the addition of IL-4 may enhance the immunogenicity of the vaccine and boost the humoral immune response, resulting in the production of more specific antibodies.

The neutralizing antibodies against PRV and PCV2 in mouse serum were detected using a microneutralization assay. The production of neutralizing antibodies commenced between d 14-21 post-immunization and peaked at 28-35 d post-vaccination (dpv). As negative controls, mice injected with DMEM did not generate any neutralizing antibodies against PRV and PCV2 (**Figures 1E, F**).

Immunohistochemistry analysis assessed the colonization of recombinant viruses rPRV-2Cap/3Cap and rPRV-2Cap/3Cap/IL4 in mouse spleens. The results depicted in **Figure 1G** demonstrate that rPRV-2Cap/3Cap and rPRV-2Cap/3Cap/IL4 could colonize the spleen of mice, whereas no detectable colonization was observed in the control group mice.

### Cellular immunity

**Figure 5:**
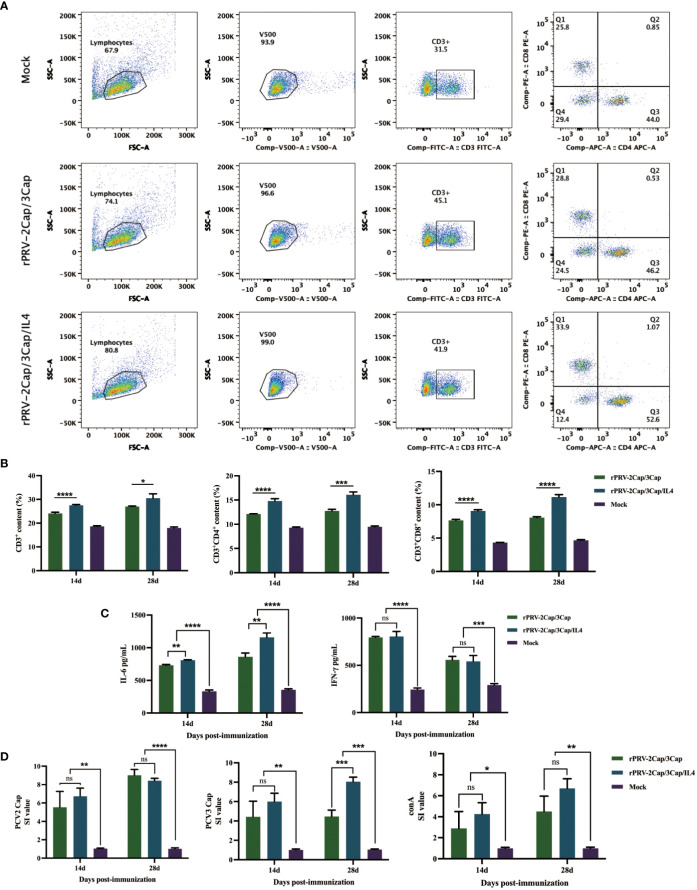
Cell immune responses after muscular immunization with recombinant virus. **(A, B)** Representative gating strategy for T cells and the flow cytometry analysis result of CD3^+^, CD3^+^CD4^+^, and CD3^+^CD8^+^ T cell populations at 14 dpv after vaccination. **(C)** The serum IL-6 and IFN-γ were detected by ELISA. **(D)** Splenic lymphocyte-specific proliferative responses assay. Data are presented as mean ± SD (n=3); ns, not significant; *, *P*<0.5; **, *P <*0.01; ***, *P <*0.001; ****, *P*<0.0001.

The changes in the numbers of CD3^+^CD4^+^ and CD3^+^CD8^+^ T lymphocytes in mouse splenocytes were assessed by flow cytometry to evaluate the status of the immune response in the body. As shown in **Figures 5A, B**, measurements were taken on the spleen of mice in the second week after immunization with enhanced rPRV-2Cap/3Cap and rPRV-2Cap/3Cap/IL4. It was found that the numbers of CD3^+^CD4^+^ and CD3^+^CD8^+^ T lymphocytes were significantly higher compared to the control group, indicating that the recombinant vaccine-induced cellular immunity in mice.

Cytokine detection kits were used to measure the secretion levels of IL-6 and IFN-γ at 14 and 28 d after immunization. The results showed that, compared to the control group, both rPRV-2Cap/3Cap and rPRV-2Cap/3Cap/IL4 promoted the secretion of IFN-γ and IL-6 in mice (**Figure 5C**), indicating that both recombinant viruses elicited a robust cellular immune response in the body. In particular, the rPRV-2Cap/3Cap/IL4 group exhibited significantly higher IL-6 secretion at 28 d post-immunization compared to rPRV-2Cap/3Cap (*P*<0.01).

To assess the *in vitro* proliferative capacity of T lymphocytes in the spleen of the experimental mice, lymphocytes were isolated from mouse spleens at 14 and 28 d post-immunization for specific lymphocyte proliferation assays. By stimulating with purified PCV2 Cap, PCV3 Cap proteins, and ConA, the differences in the *in vitro* proliferation indices (SI values) of spleen cells from mice in the rPRV-2Cap/3Cap and rPRV-2Cap/3Cap/IL4 immunized groups compared to the control group were compared (**Figure 5D**). The results demonstrated that immunization of mice with recombinant viruses, rPRV-2Cap/3Cap and rPRV-2Cap/3Cap/IL4, significantly enhanced lymphocyte proliferation, thereby promoting the occurrence of cellular immune responses.

### Mouse challenge

**Figure 6:**
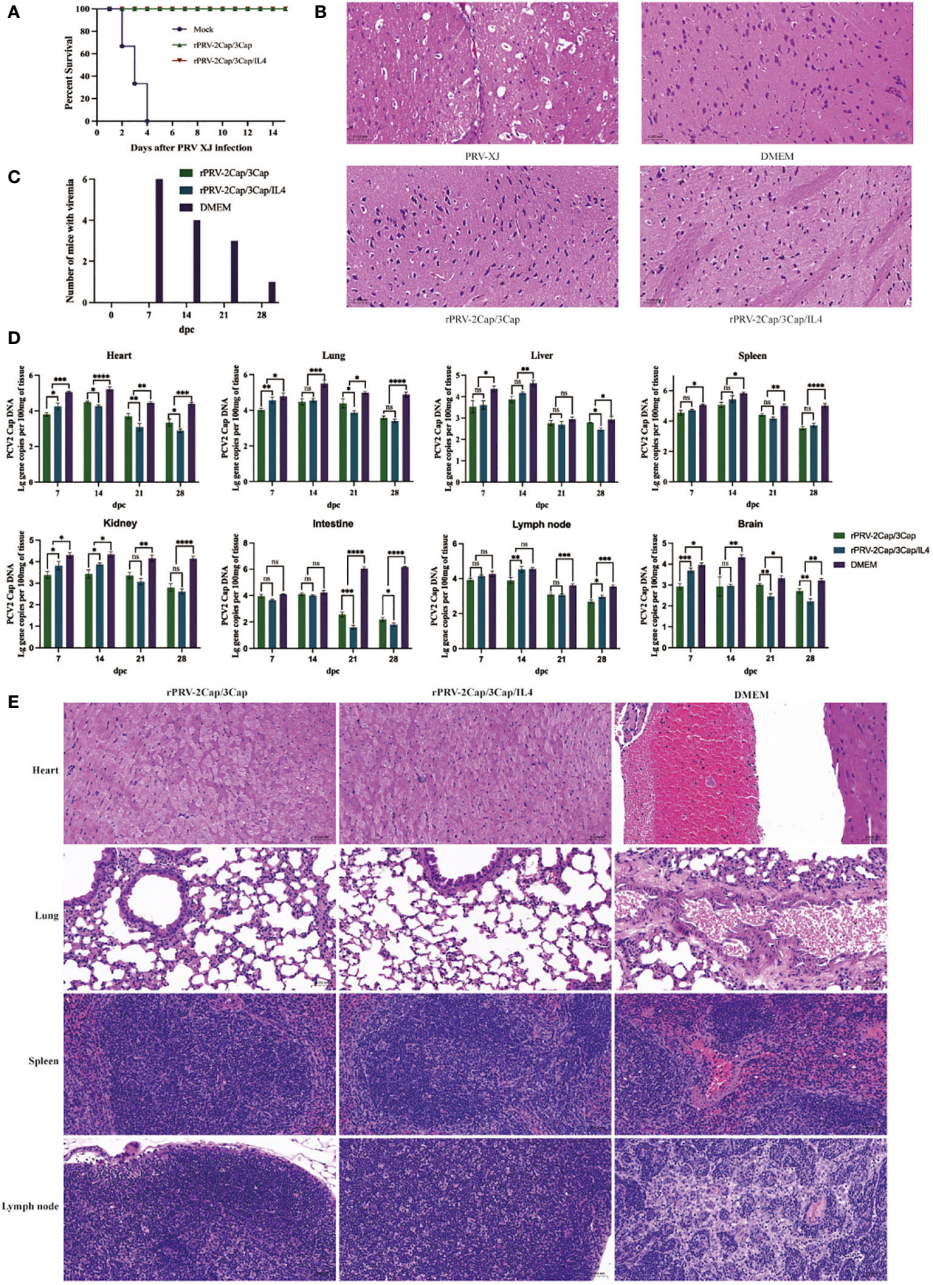
rPRV-2Cap/3Cap and rPRV-2Cap/3Cap/IL4 help mice against PRV-XJ and PCV2 infection. **(A)** The survival curves of mice challenged with the PRV-XJ in rPRV-2Cap/3Cap, rPRV-2Cap/3Cap/IL4, and DMEM groups. **(B)** Pathological observation of H&E staining in brain tissue of mice in each group. **(C)** Changes in viremia in each group from week 1 to week 4 after the PCV2 challenge. **(D)** Comparation of PCV2 Cap gene copies in heart, lung, liver, spleen, kidney, intestine, lymph node, and brain tissue of mice in each group. **(E)** Pathological observation of H&E staining in mice’s heart, lung, spleen, and lymph node tissue in each group. In the DMEM group, myocardium and lung tissues exhibited conspicuous hemorrhage, pulmonary interstitial thickening, minimal spleen and lymph nodes bleeding, and reduced lymphocytes within the lymph nodes, accompanied by infiltration of inflammatory cells. The recombinant virus group showed no apparent pathological damage. Data are presented as mean ± SD (n=3); ns, not significant; *, *P*<0.5; **, *P*<0.01; ***, *P*<0.001; ****, *P*<0.0001.

At the 8th week post-primary immunization, mice from each group were subjected to a PRV challenge with a 200 ml 10^5^ TCID_50_ dose. In the control group, mice started to die on the 2nd d, and all died by the 4th d, exhibiting typical neurological symptoms and itching before death. However, mice immunized with the recombinant viruses showed 100% protection against PRV attack (**Figure 6A**), and there were no pathological changes in the brain (**Figure 6B**).

Viral viremia and tissue viral load were evaluated in mice before (d 0) and at 7, 14, 21, and 28 d after the PCV2 challenge. Mice immunized with recombinant viruses rPRV-2Cap/3Cap and rPRV-2Cap/3Cap/IL4 showed no detectable viremia after challenge (**Figure 6C**). In contrast, in the DMEM control group, all mice had detectable viremia at 7 d after the challenge, 50% of mice had detectable viremia at 21 d after the challenge, and 17% of mice still had detectable viremia at 28 d after the challenge. Additionally, 28 d after the challenge, tissue viral loads in all organs were lower in the immunized groups compared to the DMEM control group (*P*<0.01), with reductions observed in the heart, lung, kidney, and small intestine (**Figure 6D**).

Following the PCV2 challenge, mice in the DMEM group exhibited pathological changes in cardiac tissue characterized by myocardial hemorrhage, pulmonary manifestations of interstitial thickening and pulmonary hemorrhage, spleen with minor bleeding, and lymph nodes with reduced lymphocytes, inflammatory cell infiltration, and minor bleeding. In contrast, no significant histopathological changes were observed in the tissues of the rPRV-2Cap/3Cap and rPRV-2Cap/3Cap/IL4 groups (**Figure 6E**).

## Discussion

PCV2 and PCV3 are two species of porcine circoviruses that have garnered significant attention in the current research. Due to their wide dissemination, they have become major public health and livestock industry concerns. Vaccination is recognized as an effective measure for preventing and controlling PCV. However, PCV3 is a novel virus, and its association with diseases is still under investigation. So far, no commercially available PCV3 vaccine has been produced, indicating a need for advancements in this field.

The circovirus Cap protein exhibits high immunogenicity and is one of the key factors in inducing host immunogenicity. Additionally, it can induce apoptosis and inflammatory responses in host cells, further exacerbating the severity of viral infections (26). Furthermore, the Cap protein possesses distinct immunological active regions, including epitopes that can induce T-cell and B-cell immune responses (27, 28). These epitopes may serve as targets for vaccine design to enhance immune protection against porcine circovirus infection.

Currently, recombinant viral vector vaccines are a hot topic in vaccine research. Viruses such as vaccinia virus, adenovirus, and herpesvirus are used as templates for developing viral vector vaccines (29). Herpesvirus has a robust genome and clear genetic background, making it widely applied in developing viral vector vaccines. Pseudorabies virus (PRV) is a type of herpesvirus, and the thymidine kinase (TK) gene is an important nonessential replication virulence gene, often serving as a major deletion target in viral vector vaccine research. Studies have shown that PRV strains lacking the TK gene can significantly reduce pathogenicity in mice (30) and stimulate higher levels of antibody production in pigs (31). In addition, the gE and gI genes are commonly present as a non-covalent complex, and their deletion results in a significant reduction in the virulence of PRV without affecting its replicative capacity (32). Moreover, due to the high conservation of the gE gene, it is also suitable as a molecular diagnostic marker for distinguishing vaccine strains from field strains.

The administration of PCV2 vaccines can induce cellular and humoral immunity in pigs against PCV2, thereby reducing PCV2 infection and viremia and effectively preventing clinical symptoms and tissue pathological damage caused by PCV2 (33). Specific antibody levels and neutralizing antibody levels are important criteria for evaluating the humoral immune response of vaccines. After immunizing mice with the rPRV-2Cap/3Cap and rPRV-2Cap/3Cap/IL4 constructs developed in this study, high levels of specific antibodies against PCV2 and PCV3, as well as PCV2 neutralizing antibodies, were generated. Moreover, the rPRV-2Cap/3Cap/IL4 group exhibited significantly higher levels of specific IgG antibodies compared to the rPRV-2Cap/3Cap group, indicating that IL-4 can effectively promote B cell proliferation, thereby enhancing the humoral immune response and inducing the production of high levels of specific IgG antibodies. In other studies, researchers inserted the PCV2 ORF2 or PCV3 ORF2 genes into an adenovirus vector to construct a live vector vaccine. After immunization, specific antibodies against PCV2 or PCV3 were significantly higher than in the control group, indicating a robust humoral immune response (20, 34), consistent with our findings, where Cap protein induced effective humoral immune responses when immunizing mice using various live vectors.

Cytokine detection, lymphocyte proliferation assays, and flow cytometry are important indicators for evaluating the cellular immune response of vaccines. To effectively induce adaptive immunity in the host, activation of the innate immune system is required (35). Cytokines play a key role in the innate immune response, enhancing adaptive immune responses. The levels of Th1 and Th2 cytokines are important indicators of cellular immunity, and thus, in this study, IFN-γ and IL-6 were selected for detection. IFN-γ, as a Th1 cytokine, directly reflects the regulatory level of cellular immunity. In the experiment, the immunization group showed a highly significant difference in IFN-γ levels compared to the control group (*P*<0.001), indicating that the recombinant vaccine effectively upregulates cellular immunity in mice. On the other hand, IL-6, as a Th2 cytokine, primarily reflects the activation level of B cells. In addition, IL-6 is mainly produced by mononuclear macrophages, and its level can also indirectly reflect the proliferation and function of macrophages after initial immunization. In the experiment, the levels of IL-6 were higher in the rPRV-2Cap/3Cap/IL4 group of mice, suggesting that the addition of IL-4 in the vaccine effectively promotes polarization of Th2 cells and consequently upregulates the proliferation, differentiation, and production of IgG antibodies by B cells, as well as activating the immunoregulatory function of macrophages and the production of cytokines (36).

Additionally, significant differences were observed in T lymphocyte proliferation responses between the immunized mice with the recombinant vaccine and the control group. The CD3^+^CD4^+^ and CD3^+^CD8^+^ T lymphocytes were significantly upregulated, indicating that the vaccine elicited an effective cellular immune response. Furthermore, the numbers of CD3^+^CD4^+^ and CD3^+^CD8^+^ T lymphocytes in the rPRV-2Cap/3Cap/IL4 group were significantly higher than those in the rPRV-2Cap/3Cap group, as well as the lymphocyte proliferation levels induced by PCV3, suggesting that IL-4 also can promote T cell proliferation, thereby enhancing the induction of cellular immunity.

The protective effect measures the effectiveness of a vaccine against subsequent pathogen challenges. In this study, mice were immunized with rPRV-2Cap/3Cap and rPRV-2Cap/3Cap/IL4 vaccines. When exposed to the parental strain PRV XJ, all vaccinated groups achieved 100% protection. In the PCV2 SC strain challenge experiment, the control group exhibited significant viral viremia, while the vaccinated mice did not develop viral viremia. This result indicated that the recombinant viral vaccine effectively reduces the occurrence of viral viremia. Simultaneously, the vaccinated groups exhibited lower tissue viral loads than the control group, indicating the recombinant virus’s suppression of PCV2 colonization in the host.

Additionally, histopathological observations revealed that the control group displayed more severe pathological damage, primarily manifested in lesions in the heart, lungs, spleen, and lymph nodes. Previous studies have demonstrated that PCV2 induces damage to the lungs, spleen, and lymph nodes in mice, which is consistent with the findings of this study (37). While in this experiment, no significant pathological damage was observed in mice from both the rPRV-2Cap/3Cap/IL4 group and the rPRV-2Cap/3Cap group, indicating that the recombinant virus provides effective protection against pathological damage in mice.

PCV2, PCV3, and PRV are serious infectious diseases the global pig industry faces. They are widely spread in pig farms in our country, and the co-infection rate of PCV2 and PCV3 has been increasing yearly, leading to significant losses in the pig industry in China. Vaccination plays a crucial role in preventing and controlling various emerging infectious diseases. Recombinant viral vaccines play a key role in the development of novel vaccines. In this experiment, a pseudorabies virus recombinant vaccine expressing PCV2 Cap, PCV3 Cap, and IL-4 was constructed. The vaccine demonstrated good safety and immunogenicity in a mouse model, providing protective immunity against PRV and PCV2 infection. Although no challenging experiment was conducted for PCV3, this study provides preliminary evidence of its potential as a preventive vaccine against PCV2 and PCV3 and pseudorabies virus vector vaccines. Moreover, it confirms that IL-4, as a vaccine adjuvant, can effectively enhance the humoral immune response, improve vaccine protection efficacy, and serve as a basis for developing effective bivalent recombinant vaccines against circovirus diseases.

## Data availability statement

The datasets presented in this study can be found in online repositories. The names of the repository/repositories and accession number(s) can be found in the article/supplementary material.

## Ethics statement

The animal study was approved by Sichuan Agriculture University Animal Care and Use Committee (license number SCXK (Sichuan) 2013-0001). The study was conducted in accordance with the local legislation and institutional requirements.

## Author contributions

YY: Writing – original draft, Writing – review & editing. ZX: Writing – original draft, Funding acquisition. QT: Project administration, Writing – review & editing. LX: Software, Writing – review & editing. SG: Methodology, Writing – review & editing. YH: Methodology, Writing – review & editing. ZL: Supervision, Writing – review & editing. YZ: Data curation, Writing – review & editing. JW: Formal analysis, Writing – review & editing. SL: Validation, Writing – review & editing. LZ: Methodology, Writing – original draft.
